# The complete mitochondrial genome of *Soriculus nigrescens* (Soricomorpha: Soricinae)

**DOI:** 10.1080/23802359.2019.1644557

**Published:** 2019-07-23

**Authors:** Haijun Jiang, Wanlu Deng, Haixue Wei, Qiong Wang, Shunde Chen

**Affiliations:** College of Life Sciences, Sichuan Normal University, Chengdu, China

**Keywords:** Eulipotyphla, Himalayan Shrew, phylogenetic relationship

## Abstract

The Himalayan Shrew (*Soriculus nigrescens*) Gray, 1842 belongs to the subfamily Soricinae, which is distributed in southwest China, Nepal, India, and Bhutan. This species is classified as Least Concern on The IUCN Red List. In this study, we sequenced the complete mitochondrial genome of *S. nigrescens*. This mitogenome is 17,284 bp in length and contains a set of 13 protein-coding genes (PCGs), two ribosomal RNA genes (rRNA), 22 transfer RNA genes (tRNA), one origin of L strand replication, and one control region. In order to explore the molecular phylogenetics evolution of Soricinae, the nucleotide sequence data of 13 PCGs of *S. nigrescens* and other 17 Insectivores were used for the phylogenetic analysis.

The Soricidae has 448 species in 26 genera (Burgin and He 2018) while the Soricinae has 148 species in Asia, Europe, and North America (Smith and Xie 2009). In China, there are 39 species (Smith and Xie 2009). The Himalayan Shrew (*Soriculus nigrescens*) Gray, 1842 belongs to the Soricinae, which is distributed in southwest China, Nepal, India, and Bhutan (Smith and Xie 2009). This species is classified as Least Concern on The IUCN Red List (Burgin and He 2018). An individual is collected from Nyalam County of Tibet (Latitude: 28°05′11.47″N, Longitude: 86°02′41.72″E). The specimen (GB0811005) is stored in Sichuan Academy of Forestry.

In this study, we sequence the complete mitochondrial genome of *S. nigrescens*. This mitogenome is 17,284 bp in length and has been deposited in GenBank under the accession number KY454853. The whole mitochondrial genome contains a set of 13 protein-coding genes (PCGs), two ribosomal RNA genes (rRNA), 22 transfer RNA genes (tRNA), one origin of L strand replication, and one control region. The arrangement of the multiple genes is in line with other Soricidae species (Chen et al. 2015; Huang et al. 2016; Xu et al. 2016). Most genes are transcribed on the heavy (H) strand, except for the nicotinamide adenine dinucleotide dehydrogenase subunit 6 (ND6) and eight tRNA genes (*tRNA-Gln*, *Ala*, *Asn*, *Cys*, *Tyr*, *Ser*, *Glu*, and *Pro*) which are transcribed on the light (L) strand. The length of tRNA genes varies from 59 to 73 bp. All PCGs start with an ATG codon except *ND2* that starts with ATA. Eight protein-coding genes terminate with TAA whereas the *Cytb* gene terminates with AGA. The incomplete stop codons (T++ or TA+) are used in *ND1*, *ND2*, *COX3*, and *ND4*. The control region is 1823 bp in length and locates between *tRNA-Pro* and *tRNA-Phe* genes. The total base composition mitochondrial genome is A (33.3%), C (24.3%), T (29.3%), and G (13.2%).

In order to explore the molecular phylogenetics evolution of Soricinae, the nucleotide sequence data of 13 PCGs of *S. nigrescens* and other 17 insectivores were used for the phylogenetic analysis. After alignment, the sequence dataset contained 11,417 bp. We used BEAST v1.7.4 (Drummond and Rambaut 2007) for Bayesian phylogenetic reconstruction. The best-fit GTR + I + G model of DNA substitution was obtained using J ModelTest v2 (Darriba et al. 2012) under the Akaike Information Criterion (AIC). *Suncus murinus* was used as outgroup.

Tree constructed using Bayesian phylogenetic analysis is shown in Figure 1. Bayesian analyses suggested that *S. nigrescens* and *Episoriculus caudatus* were the closest relatives as *Episoriculus caudatus* used to belong to genus *Soriculus* (Smith and Xie 2009). The complete mitochondrial genome of *S. nigrescens* will be helpful for species delimitation, phylogenetic analysis, and other relative studies in the future.

**Figure 1.**
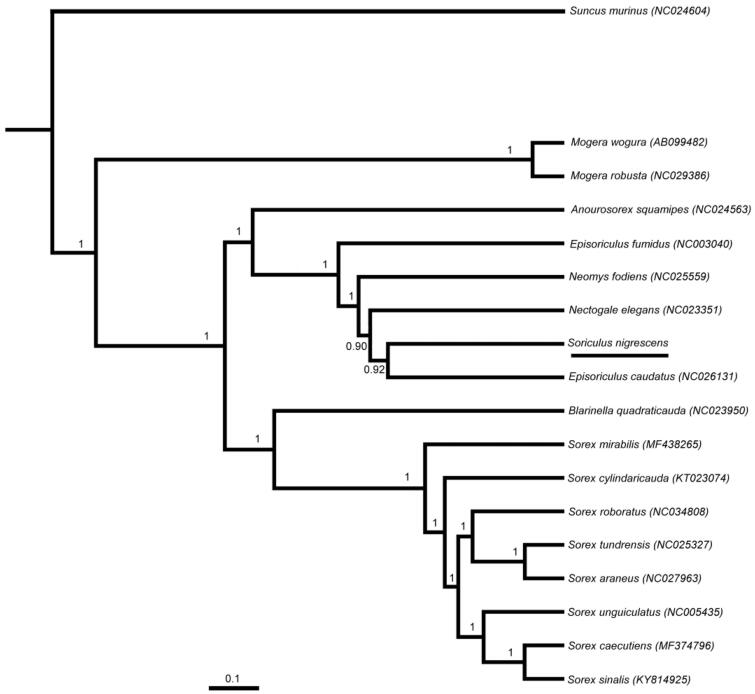
Bayesian phylogenetic analyses for *Soriculus nigrescens* based on complete mitochondrial genome
